# Epigenetic modifications and glucocorticoid sensitivity in Myalgic Encephalomyelitis/Chronic Fatigue Syndrome (ME/CFS)

**DOI:** 10.1186/s12920-017-0248-3

**Published:** 2017-02-23

**Authors:** Wilfred C. de Vega, Santiago Herrera, Suzanne D. Vernon, Patrick O. McGowan

**Affiliations:** 1 Department of Biological Sciences, University of Toronto, Scarborough, 1265 Military Trail, Toronto, ON M1C 1A4 Canada; 2 Department of Cell and Systems Biology, University of Toronto, Toronto, ON Canada; 3 Department of Psychology, University of Toronto, Toronto, ON Canada; 4 Department of Physiology, Faculty of Medicine, University of Toronto, Toronto, ON Canada; 5 Solve ME/CFS Initiative, Los Angeles, CA USA; 6 Present affiliation: Department of Biological Sciences, Lehigh University, Bethlehem, PA USA; 7 Present affiliation: The Bateman Horne Center of Excellence, Salt Lake City, UT USA

**Keywords:** Chronic fatigue syndrome, Myalgic encephalomyelitis, Epigenetics, Dna methylation, Glucocorticoid, Hpa axis, Immune cells

## Abstract

**Background:**

Myalgic Encephalomyelitis/Chronic Fatigue Syndrome (ME/CFS) is a debilitating idiopathic disease characterized by unexplained fatigue that fails to resolve with sufficient rest. Diagnosis is based on a list of symptoms and exclusion of other fatigue-related health conditions. Despite a heterogeneous patient population, immune and hypothalamic-pituitary-adrenal (HPA) axis function differences, such as enhanced negative feedback to glucocorticoids, are recurring findings in ME/CFS studies. Epigenetic modifications, such as CpG methylation, are known to regulate long-term phenotypic differences and previous work by our group found DNA methylome differences in ME/CFS, however the relationship between DNA methylome modifications, clinical and functional characteristics associated with ME/CFS has not been examined.

**Methods:**

We examined the DNA methylome in peripheral blood mononuclear cells (PBMCs) of a larger cohort of female ME/CFS patients using the Illumina HumanMethylation450 BeadChip Array. In parallel to the DNA methylome analysis, we investigated in vitro glucocorticoid sensitivity differences by stimulating PBMCs with phytohaemagglutinin and suppressed growth with dexamethasone. We explored DNA methylation differences using bisulfite pyrosequencing and statistical permutation. Linear regression was implemented to discover epigenomic regions associated with self-reported quality of life and network analysis of gene ontology terms to biologically contextualize results.

**Results:**

We detected 12,608 differentially methylated sites between ME/CFS patients and healthy controls predominantly localized to cellular metabolism genes, some of which were also related to self-reported quality of life health scores. Among ME/CFS patients, glucocorticoid sensitivity was associated with differential methylation at 13 loci.

**Conclusions:**

Our results indicate DNA methylation modifications in cellular metabolism in ME/CFS despite a heterogeneous patient population, implicating these processes in immune and HPA axis dysfunction in ME/CFS. Modifications to epigenetic loci associated with differences in glucocorticoid sensitivity may be important as biomarkers for future clinical testing. Overall, these findings align with recent ME/CFS work that point towards impairment in cellular energy production in this patient population.

**Electronic supplementary material:**

The online version of this article (doi:10.1186/s12920-017-0248-3) contains supplementary material, which is available to authorized users.

## Background

Myalgic Encephalomyelitis/Chronic Fatigue Syndrome (ME/CFS) is an idiopathic disease characterized by profound and debilitating fatigue, cognitive impairment, unrefreshing sleep, autonomic manifestations and post-exertional malaise [1]. Other known diseases or health conditions that could explain the persistent presence of fatigue, such as major depression, anorexia, and bulimia nervosa are excluded prior to ME/CFS diagnosis. Resulting heterogeneity in the clinical features of ME/CFS is an obstacle to determine its biological basis.

Many studies examining the pathophysiology of ME/CFS have reported alterations in the hypothalamic-pituitary-adrenal (HPA) axis. The HPA axis is a major component of the neuroendocrine system that regulates homeostatic processes, circadian rhythms, and environmental stress responses through a hormone cascade leading to the release of glucocorticoids (GCs). GCs interact with the GC receptor (GR) to regulate stress response and inflammation. ME/CFS patients show mild hypocortisolism and enhanced negative feedback response to GCs [2–4], suggesting a major role of the HPA axis in this disease.

In addition to modified HPA axis function, alterations in immune phenotype have been widely documented in ME/CFS. Although the specific patterns of differences remain unresolved, ME/CFS is associated with abnormal cytokine profiles [5, 6], lymphocyte proportions [7–9] and impaired immune functioning, notably decreased cytotoxicity [10–12]. Increased inflammation in the gut microbiome has also been associated with ME/CFS. These include reduced gut microbiome diversity, shifts towards pro-inflammatory bacterial species, and a proliferation of markers of pro-inflammatory processes in the serum [13].

Epigenetic modifications, including the methylation of DNA at CpG dinucleotides, can influence phenotypic changes in a long-term manner in response to external stimuli. DNA methylation modifications in genes involved in the HPA axis and the immune systems have been strongly linked to environmental stress conditions [14, 15]. We previously documented DNA methylome abnormalities in peripheral blood mononuclear cells (PBMCs) from sudden-onset ME/CFS patients, which were validated with bisulfite pyrosequencing [16]; these abnormalities were significantly concentrated in genes linked to immune regulation. Key questions remain as whether these epigenetic modifications impact immune cell function and their relationship to clinical features of ME/CFS.

In the present study, we mapped loci that were epigenetically modified in PBMCs and examined their sensitivity to glucocorticoids. Our goals were to determine how epigenetic patterns relate to HPA axis signaling in immune cells in ME/CFS patients, and to identify neuroimmune pathways impacted by ME/CFS.

## Methods

### Subject selection criteria

A pool of 231 ME/CFS diagnosed and healthy volunteers at 4 clinical sites in the USA was recruited by the SolveCFS Biobank. ME/CFS was diagnosed based on the Fukuda and Canadian criteria [1, 17]. Each volunteer answered surveys about symptoms, medication use, and medical history and completed the RAND-36 self-reported survey [18] to assess health-related quality of life. ME/CFS appears to be up to 1.5 times more likely to affect females [19]. We therefore specifically selected females for this study. From the volunteer pool, 49 ME/CFS patients and 25 healthy controls met the following criteria: 1) tested negative for HIV, AIDS, and/or Hepatitis C and 2) were white non-obese females (BMI < 30) with no prior history of immunomodulatory and/or epigenetic-active medication consumption. The latter criterion was aimed at minimizing potential confounding effects on the DNA methylome and immune response.

### PBMC isolation and storage

Whole blood from each volunteer was collected in sodium heparin tubes and shipped overnight at ambient temperature room temperature to the Rutgers University’s Cell and DNA Repository where they were processed. Briefly, Peripheral Blood Mononuclear Cells (PBMCs) were isolated by Ficoll gradient centrifugation and resuspended in 1X Dulbecco’s phosphate-buffered saline (DPBS) + 1% fetal bovine serum (FBS). Cell count estimates were obtained using a ViCell XR Viability Analyzer. After counting, approximately 10×10^6^ PBMCs were pelletized through centrifugation, dried and stored at -80 °C. The remaining PBMCs were cryopreserved in 10% dimethyl sulfoxide (DMSO), 50% FBS, and 40% Roswell Park Memorial Institute 1640 medium (RPMI-1640), distributed in 1 ml aliquots, and stored in liquid nitrogen. PBMC cell pellets and cryopreserved PBMCs were shipped on dry ice to the University of Toronto for subsequent analyses.

### Genomic DNA extraction and purification

To obtain purified genomic DNA from the 49 ME/CFS patients and 25 healthy controls, we used the Omega E.Z.N.A. Tissue DNA kit following manufacturer’s instructions (Omega Bio-Tek, cat. no. D3396) on a sample of approximately 2.50×10^6^ PBMCs from dry pellets by fractioning. DNA was eluted in Tris-EDTA buffer (10 mM Tris-CL, pH 8.5, 1 mM EDTA). We quantified its purity and concentration using a NanoDrop 2000c Spectrophotometer (Thermo Scientific, Waltham, MA, USA). Elutions were further purified using the Qiagen MinElute Reaction Cleanup Kit (Qiagen Canada, cat. no. 28204) when DNA purity did not meet standard absorbance criteria, i.e., A_260_/A_280_ = 1.8-2.0, and A_260_/A_230_ > 2.0. We diluted the purified DNA to a final concentration of approximately 100 ng/μl.

### DNA methylome arrays

We used the Illumina Infinium HumanMethylation450 BeadChip (450 K) array (Genome Québec core facility, Montreal, QC) to obtain DNA methylome profiles from ME/CFS patients (*n* = 49) and healthy controls (*n* = 25). Approximately 1.5 μg of purified genomic DNA from each individual was bisulfite converted using the EZ DNA Methylation Kit (Zymo Research) and subsequently analyzed following standard Illumina protocols for the 450 K platform. The 450 K array interrogates the methylation levels of more than 480 000 CpG loci, which cover 99% of RefSeq Genes and 96% of CpG islands in the human genome. All the 450 K raw data from this project have been deposited in the Gene Expression Omnibus (GEO) database of the US National Center for Biotechnology Information NCBI under the accession number GSE93266.

### DNA methylome data normalization and statistical analyses

DNA methylome profile analysis was performed in R using the Illumina Methylation Analyzer (IMA) package [20] and Minfi [21]. Data from each 450 K array were annotated according to the Human Genome Build 37 available at the UCSC Genome Browser (http://genome.ucsc.edu/). Raw probe florescence intensities were normalized by Subset-quantile Within Array Normalization (SWAN) [22]. Methylation-level values for each CpG site were estimated as beta-values. A beta value is defined as the ratio of methylated probe fluorescence intensity over total intensity (methylated plus unmethylated probe intensities). Beta-values range from 0 to 1 and are equivalent to the percentage of methylation of the CpG site [23]. DNA methylation differences on the 450 K array are known to either be confounded due to genetic polymorphisms or masked due to the large presence of invariably methylated sites in the genome [24, 25]. To optimize the number of significant DNA methylation calls, we discarded loci that met the following criteria: 1) the fluorescence intensity signal of the probe in the array was statistically indistinguishable from background (detection *p*-value ≤ 0.01); 2) contained SNPs, according to dbSNP versions 132, 135, and 137, either at the interrogated CpG locus or at the flanking single nucleotide extension; 3) were invariable across samples with respect to methylation (i.e., mean beta-value ≥ 0.95 or ≤ 0.05). To account for epigenetic variation that may arise from confounding factors, we corrected the beta-values for batch effects using the ComBat algorithm [26], and included age, Body Mass Index (BMI), and estimated cell compositions as covariates [27]. Differentially methylated sites were identified using the Wilcoxon-rank sum test. Benjamini-Hochberg procedure/false discovery rate (FDR) was used to correct for multiple testing. We considered loci as differentially methylated, when comparing ME/CFS patients with healthy controls, if they met the all of the following criteria: 1) mean beta-difference of ≥ 0.05; 2) nominal Wilcoxon-rank sum test *p*-value ≤ 0.05; and 3) FDR-corrected *p*-value of ≤ 0.05. In addition, we performed a Pearson Chi-Squared Test in R to compare differences in proportion of differential methylation according to genic region and distance from a known CpG island. To further evaluate the significance of association between methylation beta-values in each locus and dexamethasone suppression assay subgroups, we performed non-parametric permutation tests in R. To do so, we generated null distributions of the mean beta-difference per locus by: 1) Randomly reordering the dexamethasone suppression assay subgroup assignments in each comparison (i.e., control vs. ME/CFS GC-Hypersensitive, control vs. ME/CFS GC-Typical, ME/CFS GC-Hypersensitive vs. ME/CFS GC-Typical); 2) Calculating the mean beta-difference per probe; and 3) Repeating steps 1 and 2 10,000 times. Approximate p-values for each randomization test were calculated as the proportion of mean beta-difference values in the generated null distribution that were equal or more extreme than the observed value for each probe.

To identify potential functions and cellular locations of genes associated with differentially methylated loci, we performed a Gene Ontology (GO) analysis using the program DAVID [28, 29]. Enrichment Map [30] was used to cluster GO terms according to the amount of gene overlap and were textually summarized using the WordCloud plugin.

### DNA methylation validation by bisulfite pyrosequencing

We used a nested primer design to enhance amplification of regions targeted for methylation analysis by bisulfite pyrosequencing. First, ‘nested’ bisulfite pyrosequencing assays for the loci of interest were designed using the Qiagen PyroMark Assay Design Software 2.0. Additional ‘outside’ primers targeting regions that encapsulated those targeted by the PyroMark assay designs were designed using Primer3 [31, 32]. Genomic DNA (300 ng) was bisulfite converted using the Zymo EZ DNA Methylation-Gold Kit according to the manufacturer’s instructions. After bisulfite conversion, 15 ng of bisulfite converted DNA was subjected to PCR to obtain biotinylated products for pyrosequencing. Each sample was amplified with 200 μM of dNTPs, 200 nM of forward and reverse primer (listed in Table 3), and 0.625 units of NEB Thermopol Taq Polymerase. The thermocycling protocol for the outside PCR was: 1 cycle of 95 °C/30 s; 30 cycles of 95 °C/30 s, 57 °C/30 s, and 68 °C/30 s; and 1 cycle of 68 °C/5 min. The thermocycling protocol for the nested PCR was: 1 cycle of 95 °C/30 s; 30 cycles of 95 °C/30 s, 53 °C/30 s, and 68 °C/30 s; and 1 cycle of 68 °C/5 min. Bisulfite pyrosequencing was performed on a Pyromark Q106 ID pyrosequencer with Pyromark Q-CpG 1.0.9 software.

### Dexamethasone suppression assay and association with DNA methylation differences

Cryopreserved PBMCs were available from ME/CFS patients (*n* = 33) and healthy controls (*n* = 24) that were also examined by DNA methylome array (see above). These cells were gently thawed in a 37 °C water bath for 5–7 min and were counted using a hemocytometer (Thermo Fisher) and assessed for viability by Trypan blue exclusion. After thawing, all samples had > 95% viability. Based on our preliminary experiments, 4.0×10^5^ live PBMCs were cultured for 4 days at 37 °C, 5% CO_2_ in RPMI-1640 + 10% fetal bovine serum [33] according to the following treatments: 1) A control treatment with cells and culture media only; 2) A stimulated treatment where cells were cultured with 5 μg/ml phytohaemagglutinin (PHA); and 3) a suppressed condition where cells were cultured with 5 μg/ml PHA and 10^-6^ M dexamethasone. Bromodeoxyuridine (BrdU) was added into each well on the 3^rd^ day of culture and cell proliferation was assessed using the Roche BrdU colorimetric ELISA kit. The percentage of inhibition was then calculated using the average values of each experimental condition with the following formula:

$$ \mathrm{Inhibition}\%=\frac{\mathrm{Stimulated}-\mathrm{Suppressed}}{\mathrm{Stimulated}}\times 100\% $$

All assays were performed in triplicate.

We used a two-tailed *t*-test test to identify between-group differences in dexamethasone response, including subgroups of ME/CFS patient based on preliminary observations of a binomial distribution in the patient data. We also performed logistic regression and Pearson correlations in R to explore if ME/CFS onset type or RAND-36 scores were associated with differences in GC sensitivity. Significant differentially methylated sites in subgroups that differed in their dexamethasone suppression response were assessed according to the following statistical criteria: 1) Mean beta-difference of ≥ 0.05; and 2) nominal Wilcoxon-rank sum test *p*-value ≤ 0.05.

### Association between clinical data and DNA methylation

Significant differentially methylated CpG sites shared between comparisons were examined to determine sites that were potentially related to glucocorticoid (GC) sensitivity and ME/CFS. To detect significant associations between health-related quality of life RAND-36 scores and DNA methylome data, we performed principal component analyses (PCA), linear regression, and FDR-correction using the stats package in R. We restricted this analysis to differentially methylated regions, defined according to the 450 K annotations and having a minimum of 2 sites with mean beta-difference ≥ 0.05, to reduce statistical noise. Two-tailed t-tests were performed on demographic information and RAND-36 scores, and a one-tailed *t*-test was performed on pyrosequencing data were compared using IBM SPSS Software (Version 22).

## Results

### RAND-36 scores are significantly lower in ME/CFS patients

Overall, ME/CFS patients had lower health-related quality of life than healthy controls, scoring significantly lower in 7 of the 8 RAND-36 categories (all p-values ≤ 0.05; Table 1). There were no differences in the average age or BMI of the clinical groups. Principal component analysis (PCA) was performed on the RAND-36 scores in order to reduce the dimensionality of the variation in scores across all categories to have a more interpretable measure of how overall score on the RAND-36 can distinguish between ME/CFS and controls. PCA showed clear a separation between ME/CFS patients and controls, with the first two principal components (PCs) explaining 85.1% of the total variation in the data (Additional file 1: Figure S1).

**Table 1:** Demographic data of ME/CFS and healthy control patients

	ME/CFS patients	Healthy control subjects
Male/Female	0/49	0/25
Age (years)	49.4 ± 1.9	51.1 ± 2.7
BMI (kg/m^2^)	23.3 ± 0.5	23.4 ± 0.6
Physical Health		
Physical Functioning	40.6 ± 3.9*	95.3 ± 1.4
Role-Physical	7.7 ± 3.1*	96.9 ± 2.2
Pain	55.9 ± 3.6*	90.0 ± 1.8
General Health	25.2 ± 2.3*	81.8 ± 2.4
Mental Health		
Energy	16.9 ± 2.3*	71.7 ± 2.5
Social Functioning	33.0 ± 3.7*	91.9 ± 2.4
Role-Emotional	71.4 ± 5.9	84.5 ± 5.4
Emotion	73.2 ± 2.4*	80.6 ± 2.6
Age ME/CFS of first symptoms (years)	31.0 ± 1.8	N/A
Age of ME/CFS diagnosis (years)	37.1 ± 1.7	N/A
Sudden/Gradual ME/CFS onset	33/16	N/A

Average demographic information and RAND-36 scores with standard error of ME/CFS and healthy controls included in this cohort. * = *p* ≤ 0.05, *t*-test

### DNA methylome differences in ME/CFS

We observed 12,608 significant differentially methylated loci in our cohort after correcting for age, BMI, and differences in cell proportions (Additional file 2: Table S1). For 5,544 of these loci, the probe was annotated to a known protein coding gene according to the UCSC Genome Browser, indicating they were associated with genes. The top 5 hypo- and hypermethylated sites according to the magnitude of methylation differences are listed in Table 2. In terms of the direction of methylation differences in ME/CFS patients compared to healthy controls, 71.6% of the differentially methylated loci were hypermethylated and 28.4% were hypomethylated (Additional file 3: Figure S2A). We categorized probes according to their annotated genic locations and their distance from a known CpG island in order to determine if differential methylation was enriched at particular regions in the genome. While there were no significant differences in proportion of hypo-/hypermethylated sites according to genic location (Additional file 3: Figure S2B), there were significant differences in these proportions when considering distance from a CpG island (Additional file 3: Figure S2C). The amount of hypermethylation decreased as distance increased from a CpG island (*p* < 0.05, Additional file 3: Figure S2C). Specifically, probes found in the N Shore (up to 2 kb upstream of a CpG island), both N and S Shelf regions (2–4 kb from a CpG island), and the Open Sea (outside of Shelf regions) significantly differed in proportion of hypo-/hypermethylation when compared to CpG islands (all FDR < 0.05, Additional file 3: Figure S2C). S Shelf probes also exhibited decreased hypermethylation compared to neighboring S Shore probes (FDR < 0.05, Additional file 3: Figure S2C).

**Table 2:** Top hypo- and hypermethylated sites between ME/CFS and healthy controls

Probe ID	Targeted gene symbol	Mean Beta-value (ME/CFS)	Mean Beta-value (control)	Beta-difference	*p*-value	Adjusted *p*-value (FDR)	Genic region	Relation to CpG Island
cg26341831	TMEM63A	0.352	0.504	-0.152	9.04e-4	1.05e-2	Body	Open Sea
cg00446123	LIME1	0.340	0.478	-0.138	1.17e-3	1.18e-2	TSS200	N Shore
cg27058497	RUNX3	0.314	0.450	-0.136	7.60e-4	9.88e-3	TSS200	Open Sea
cg08817540	HHLA2	0.390	0.524	-0.134	5.34e-4	8.92e-3	TSS1500	Open Sea
cg17587997	FYN	0.529	0.663	-0.134	8.66e-4	1.04e-2	5'UTR	Open Sea
cg00660167	N/A	0.754	0.581	0.174	5.58e-4	9.03e-3	N/A	N Shore
cg23189692	EIF4G1	0.652	0.486	0.166	5.10e-4	8.81e-3	Gene Body	N Shelf
cg17344770	C19orf71	0.674	0.511	0.163	2.00e-4	8.16e-3	TSS1500	Open Sea
cg07302959	FAM133B	0.664	0.503	0.161	9.04e-4	1.06e-2	Gene Body	Open Sea
cg06633438	MLLT1	0.626	0.466	0.160	1.65e-4	8.16e-3	Gene Body	Island

Top 5 hypo- and hypermethylated sites according to mean methylation difference between ME/CFS and controls

### Glucocorticoid sensitivity in PBMCs in ME/CFS subgroups

**Fig. 1:**
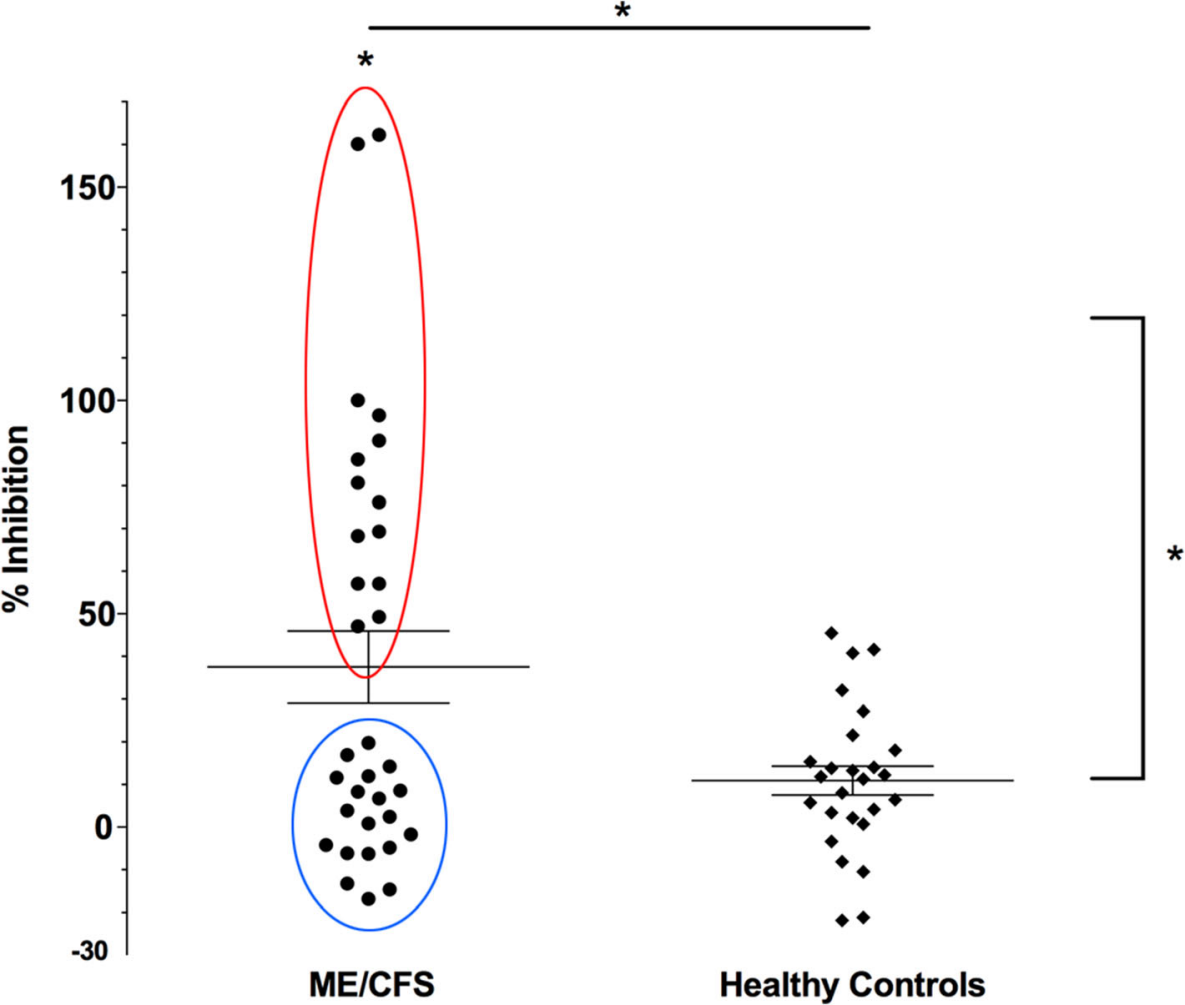
Dexamethasone suppression assay results. Dexamethasone suppression results from PBMCs of ME/CFS patients (*n* = 33) and healthy controls (*n* = 24) after stimulation with PHA. Inhibition % represents the amount of suppressed cell replication with 10^-6^ M dexamethasone compared to stimulated and unstimulated conditions. Each point represents the mean % inhibition of a single subject. The mean is represented along with standard error bars. Glucocorticoid sensitivity was greater among ME/CFS patients overall compared to healthy controls and between ME/CFS GC-Hypersensitive (*red circle*) compared to ME/CFS GC-Typical (*blue circle*; *p*’s ≤0.05)

Overall, there was a significant mean increase in glucocorticoid sensitivity in PBMCs from ME/CFS patients compared to healthy controls (*p* ≤ 0.05). A visual inspection of the data revealed a bimodal distribution of glucocorticoid sensitivity within the ME/CFS cohort: a GC-Hypersensitive group (circled in red in Fig. 1), who exhibited an increased response to glucocorticoid treatment compared to the mean control (*p* ≤ 0.05) response, and a GC-Typical group (*p* ≤ 0.05; circled in blue in Fig. 1), who exhibited a response similar to the healthy controls in our clinical cohort. Differences in GC sensitivity were not associated with type of ME/CFS onset or the RAND-36 survey when considering scores on the overall survey or in particular categories (*p* > 0.10, all comparisons).

### DNA methylation differences in dexamethasone assay subgroups via pyrosequencing and permutation tests

To determine the association between differences in DNA methylation and glucocorticoid sensitivity, we applied the same statistical criteria used to identify methylation differences between ME/CFS patients and healthy controls (see Methods) to 3 different comparisons: 1) ME/CFS GC-Hypersensitive vs. ME/CFS GC-Typical; 2) ME/CFS GC-Hypersensitive vs. Controls; and 3) ME/CFS GC-Typical vs. Controls. We found that no methylation differences met these statistical criteria. However, a large number of loci exhibited significant nominal Wilcoxon-rank sum test *p*-values ≤ 0.05. As alternative methods of examining statistical confidence in the 3 glucocorticoid sensitivity comparisons, we evaluated the differences found with the 450 K array via targeted bisulfite pyrosequencing and genome-wide permutation of the data.

**Fig. 2:**
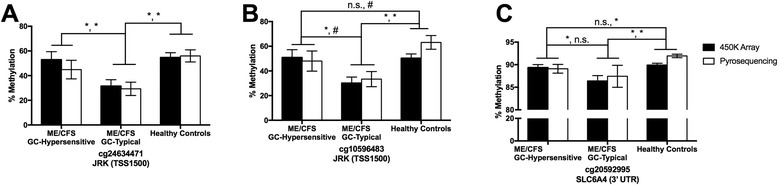
Bisulfite pyrosequencing of nominally significant sites. Pyrosequencing (*white*) results of (**a**) cg24634471, (**b**) cg10596483, and (**c**) cg20592995, sites that showed nominal significance on the 450 K array (black) when comparing means of ME/CFS GC-Hypersensitive (*n* = 14), ME/CFS GC-Typical (*n* = 19), and healthy controls (*n* = 33) assessed using an in vitro dexamethasone suppression assay. Error bars represent standard error. Lines indicate the group comparisons, where the first symbol indicates the result when compared using the 450 K array while the second statistic indicates the result compared by pyrosequencing. * = *p* ≤0.05, # = *p* ≤0.10, *t*-test

For pyrosequencing analysis, our strategy was to select 3 loci that showed nominally significant differences between ME/CFS GC-Hypersensitive and ME/CFS GC-Typical: 2 loci in JRK and 1 locus in SLC6A4. These sites were chosen to examine the reliability of the 450 K array to detect significant differences both above and below the 5% cutoff that we implemented to search for significant differentially methylated sites in ME/CFS and to specifically validate DNA methylation differences that are both potentially related to GC sensitivity differences as well as unique to ME/CFS (see Methods). The JRK sites were selected for the following reasons: these were among the top sites in terms of magnitude difference that showed a >5% methylation difference on the 450 K array, where cg24634471 showed a 21.4% difference (nominal *p* = 0.017) and cg10596483 20.8% difference (nominal *p* = 0.014) between ME/CFS GC-Hypersensitive and ME/CFS GC-Typical, and these sites were also close together in the genome (5 bp apart), which allowed us to infer how the DNA methylation status of a probe is reflected in probes within the same annotated genic region. The SLC6A4 site (cg20592995) was among the top sites showing <5% difference based on magnitude difference, and showed a 3% methylation difference (nominal *p* = 0.038) on the 450 K array between ME/CFS GC-Hypersensitive and ME/CFS GC-Typical. After pyrosequencing (primers listed in Table 3), the JRK sites were declared to be significant (*p* ≤0.05; Fig. 2a and b) or trending (*p* ≤0.10; Fig. 2B) according to pyrosequencing results. However, the <5% nominally significant difference found in the 450 K array data for SLC6A4 was not significant in the bisulfite pyrosequencing assay (Fig. 2c), indicating that methylation differences <5% on the 450 K array were not reliably detected for these comparison conditions.

**Table 3:** Bisulfite pyrosequencing primers

Targeted Gene/Site(s)	Primer Direction	Primer Sequence (5’ to 3’)
JRK (cg24634471 and cg10596483)	Out Forward	GTAGGCGGGTTGAGTATTGG
	Out Reverse	CGACCTAAACCCCGAACTCC
	In Forward	GTTTTGGTGATAGGAAGGTAGTATTGT
	In Reverse	[Biotin]-AACTCCCCCCTACTCTCTCCATCTATA
	Sequencing	GGAAGATAGTTTTGGGTTGA
SLC6A4 (cg20592995)	Out Forward	TTGGGGAAAGGAGGTTAAGG
	Out Reverse	GCTCGCTAACGATCACGATT
	In Forward	AAGTGATAGGTGGTTAGATGAT
	In Reverse	[Biotin]-CCTTTCATTTCACATAAAACCCTTAATATA
	Sequencing	TTTTTTATTTAAGTTTTTGAGAGT

Outer and inner PCR primer sequences used for the bisulfite pyrosequencing assay

For permutation analysis, we examined the amount of overlap between the sites with a >5% mean methylation difference that were nominally significant on the 450 K array according to the Wilcoxon rank-sum test and the sites that were declared to be significantly different using 10,000 permutations. We found that a majority of the nominally significant probes were also significant according to the permutation test: 76.8% in the ME/CFS GC-Hypersensitive vs. ME/CFS GC-Typical comparison, 84.5% in the ME/CFS GC-Hypersensitive vs. Control comparison, and 99.6% in the ME/CFS GC-Typical vs. Control comparison, indicating that the majority of methylation differences that were nominally significant with >5% mean methylation difference likely reflected differential methylation.

**Fig. 3:**
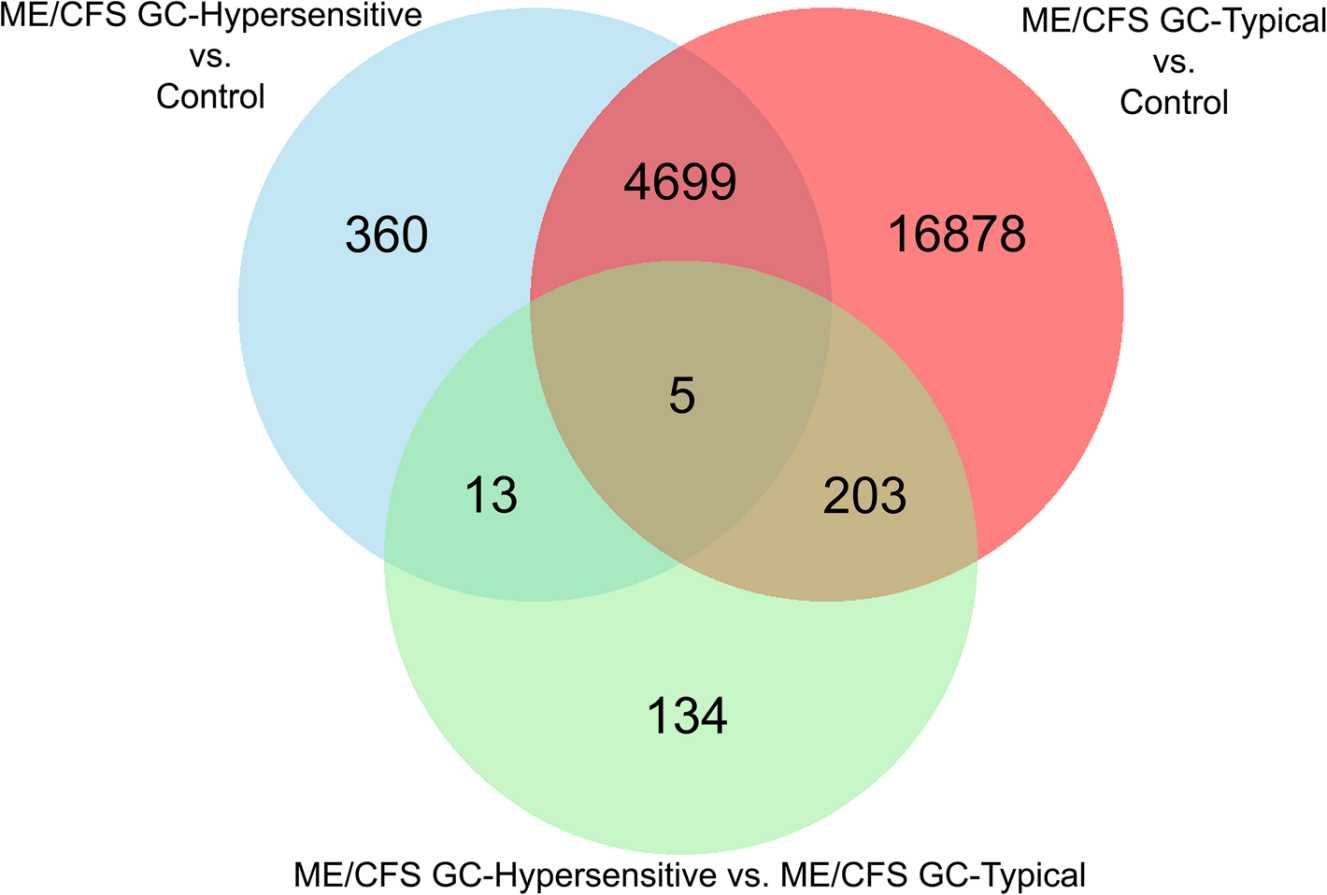
Venn diagram comparing the number of differentially methylated sites across three comparisons. Venn diagram depicting the overlap between the ME/CFS GC-Hypersensitive vs. ME/CFS GC-Typical (*green*), ME/CFS GC-Hypersensitive vs. Control (*blue*), and ME/CFS GC-Typical vs. Control (*red*) comparisons. Numbers within each circle and overlap correspond to the number of differentially methylated CpG sites

**Fig. 4:**
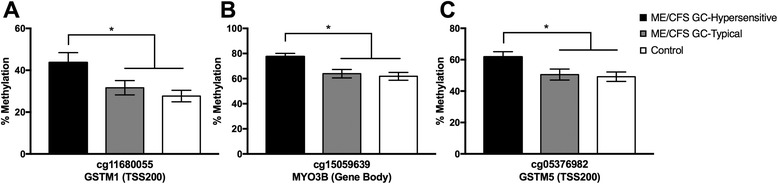
Top 3 glucocorticoid sensitive sites, based on magnitude of methylation difference. Increased methylation was observed at GpG sites of (**a**) GSTM1, (**b**) MYO3B, and (**c**) GSTM5 in the ME/CFS GC-Hypersensitive (*black*) compared to ME/CFS GC-Typical (*grey*) and Controls (*white*). * = *p* ≤0.05, Wilcoxon rank-sum test

**Fig. 5:**
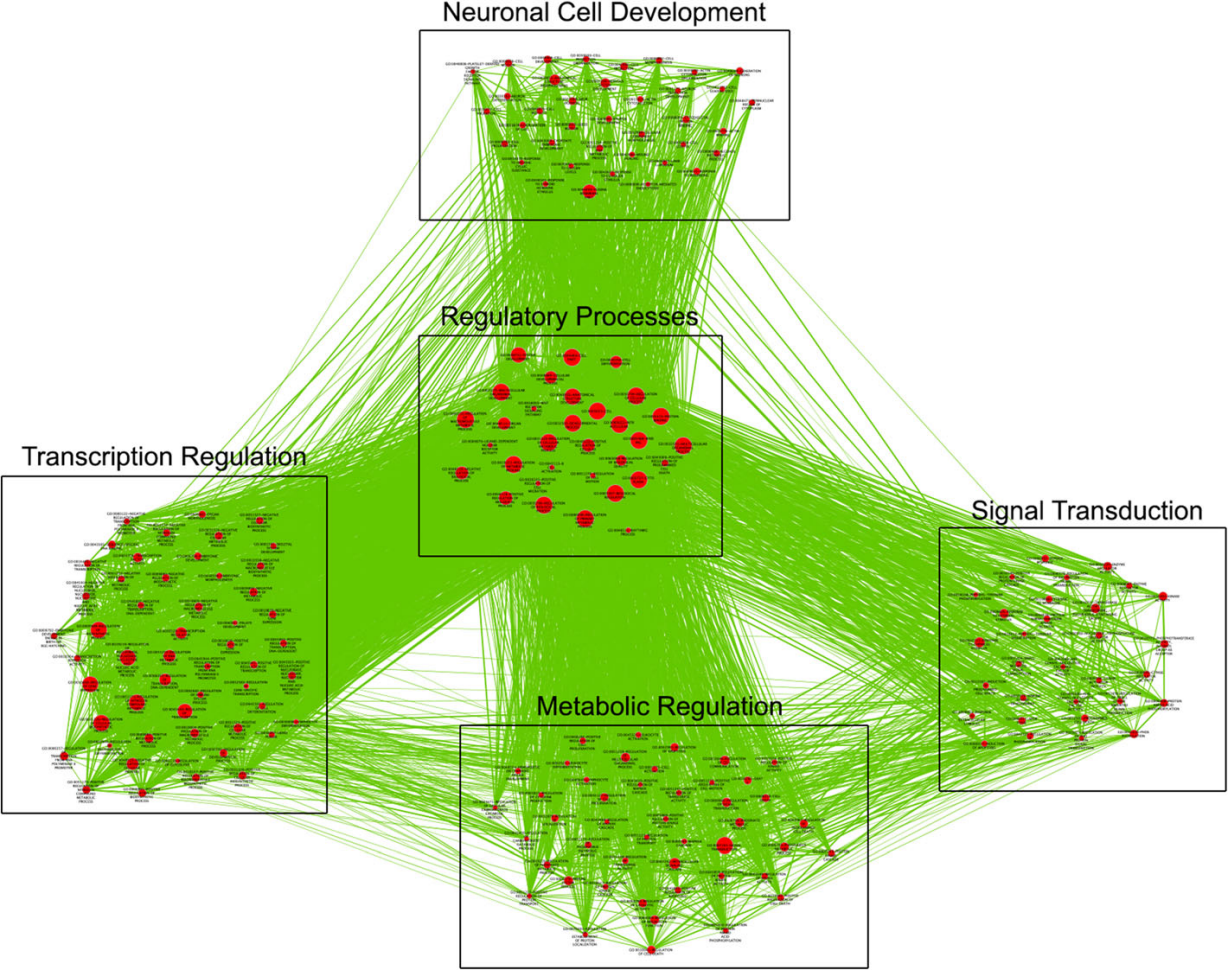
Enriched biological themes in differentially methylated sites that are likely associated with ME/CFS. A network map of 4,699 CpG sites showing significantly different methylation associated with ME/CFS, grouped according to GO terms and summary annotations (*boxes*). The size of the nodes (*red circles*) is proportional to the number of genes within each GO term and the thickness of the edges (*green lines*) represents the number of genes in common between GO terms

Given these results, we implemented the 5% difference cutoff across the various dexamethasone assay subgroup comparisons, and examined sites that were found to be significant using both the Wilcoxon rank-sum test and the permutation test. To determine potential epigenomic loci associated with glucocorticoid sensitivity, we examined the overlap in nominally significant loci across the three comparisons (Additional file 4: Table S2; Fig. 3). There were 5 sites that were differentially methylated across all 3 comparisons (Additional file 4: Table S2); one of which corresponded to a coding gene: NPAS3, a gene implicated in neurogenesis. We found 13 loci that were differentially methylated in both the ME/CFS GC-Hypersensitive vs. Controls (green) and ME/CFS GC-Hypersensitive vs. ME/CFS GC-Typical (blue) comparisons (Fig. 3). These loci, listed in Table 4 (full annotation information in Additional file 5: Table S3) with corresponding permutation results (Additional file 6: Figure S3), are likely associated with glucocorticoid sensitivity. The top 3 sites that showed the greatest magnitude of differences when comparing ME/CFS GC-Hypersensitive to GC-Typical subjects (ME/CFS and Controls) were corresponded to GSTM1 (14.3% increase in methylation), MYO3B (13.7% increase), and GSTM5 (12.0% increase; Fig. 4). In addition to these glucocorticoid sensitive sites, we found 4,699 loci likely associated with ME/CFS, as they were differentially methylated in the ME/CFS GC-Hypersensitive vs. Control (blue) and ME/CFS GC-Typical vs. Control (red) comparisons (Fig. 3, Additional file 7: Table S4). GO analysis of these sites revealed an enrichment of differential methylation in ME/CFS associated with regulatory processes, including neuronal cell development, signal transduction, metabolic regulation, and transcription regulation (Fig. 5, Additional file 7: Table S4). There were 203 significant differentially methylated sites that were unique to ME/CFS GC-Typical subjects (Fig. 3, Additional file 8: Table S5), however no GO terms were significantly associated with these sites.

**Table 4:** Epigenetic loci associated with GC sensitivity in ME/CFS

Probe ID	Targeted gene symbol	Mean Beta-value (ME/CFS GC-Hypersensitive)	Mean Beta-value (ME/CFS GC-Typical)	Mean Beta-value (control)	Genic region
cg03484234	PNPLA4	0.595	0.525	0.536	TSS1500
cg04072270	N/A	0.651	0.708	0.715	N/A
cg04438194	N/A	0.199	0.134	0.147	N/A
cg05123845	N/A	0.546	0.601	0.605	N/A
cg05252487	FAM24A	0.289	0.354	0.344	5' UTR
cg05376982	GSTM5	0.609	0.498	0.479	TSS200
cg11680055	GSTM1	0.440	0.318	0.276	TSS200
cg12042203	N/A	0.672	0.618	0.620	N/A
cg14507445	N/A	0.833	0.776	0.773	N/A
cg15059639	MYO3B	0.762	0.636	0.614	Body
cg19196401	DDO	0.520	0.615	0.598	Body
cg19251564	N/A	0.711	0.660	0.654	N/A
cg19763428	PDE1C	0.420	0.362	0.349	Body

Thirteen differentially methylated sites that are associated with glucocorticoid sensitivity in ME/CFS GC-Hypersensitive subjects

### Relationships between differentially methylated regions and health-related quality of life

To examine the association of DNA methylation with health-related quality of life, we used the first principal component (PC1), explaining 68.4% of the variance in RAND-36 scores (Additional file 1: Figure S1B), in our linear regression analysis. We examined gene regions for this analysis in order to to determine potential epigenomic regions that show a significant relationship with quality of life scores. After linear regression analysis, we found over 1,600 differentially methylated regions with a >5% methylation difference between CFS and controls that showed a significant relationship with overall RAND-36 score (Additional file 9: Table S6). The top 5 differentially methylated regions based on R^2^ are shown in Table 5, and corresponded to TSS methylation in GRAMD1A, ATP6V0E2, LOC144571, and gene body methylation in LOC401431 and IL6R.

**Table 5:** Epigenomic regions associated with quality of life

Gene	Region	R^2^	FDR	Mean (ME/CFS)	Mean (control)	Beta-difference
GRAMD1A	TSS1500	0.229	4.27e-3	0.357	0.265	0.092
ATP6V0E2	TSS200	0.226	4.27e-3	0.335	0.256	0.079
LOC401431	Gene Body	0.224	4.27e-3	0.347	0.266	0.081
IL6R	Gene Body	0.220	4.27e-3	0.380	0.304	0.076
LOC144571	TSS1500	0.220	4.27e-3	0.314	0.237	0.077

Top 5 regions based on R^2^ that show a significant relationship between DNA methylation and overall RAND-36 score

## Discussion

In this study, we detected 12,608 differentially methylated sites in PBMCs of ME/CFS patients compared to healthy controls, some of which were significantly associated with self-reported quality of life health scores. 71.6% of these sites were hypermethylated in ME/CFS and hypermethylation was found to decrease as distance from a CpG island increased, suggesting that epigenetic dysregulation in ME/CFS significantly varies depending on relative location to CpG islands. Within the ME/CFS patient group, we observed two distinct subgroups based on in vitro sensitivity to glucocorticoid exposure. The difference in glucocorticoid sensitivity was associated with differential methylation in 13 sites on the basis of comparisons between differential methylation in both ME/CFS GC-Hypersensitive compared to ME/CFS GC-Typical and ME/CFS GC-Hypersensitive compared to healthy controls.

### DNA methylation modifications in cellular processes/metabolism pathways in ME/CFS

Genes associated with cellular and metabolic regulation were major pathways showing differential epigenetic profiles in ME/CFS compared to healthy controls. These findings are consistent with a previous report by our group in sudden onset ME/CFS patients [16] and with other reports of genomic, transcriptomic, and metabolomic differences in ME/CFS [34–37], which may indicate a role for DNA methylation modifications in the metabolic stress observed in this disease. Oxidative and nitrosative stress states have been documented in immune cells from ME/CFS patients [38, 39] and a reduction in electron transport chain metabolites [37] suggests a role for processes affecting mitochondrial function in ME/CFS pathology. There is a known relationship between oxidative stress and epigenetic modifications. DNA lesions are often produced from oxidative stress states, which in turn affect the multiple levels of epigenetic regulation, leading to aberrant DNA methylation and gene expression patterns [40]. It is possible that oxidative stress, as indicated by the differences found in cellular and metabolic regulation genes in our study including ARL4C and HOXA11 (Additional file 2: Table S1; also see [41]), may drive some of the epigenetic changes observed in ME/CFS. However, additional work is required to explore this relationship, such as characterizing the effect of ARL4C and HOXA11 on DNA methylation patterns with functional genomics experiments. 

Genes associated with neuronal cell development were also a major class of genes differentially methylated in ME/CFS patients. At least two previous studies that examined gene expression patterns in PBMCs of ME/CFS patients also reported significant differences genes involving neuronal development and regulatory processes [42, 43]. It is also known that genes associated with psycho-neuroendocrine-immune pathways show rich expression profiles in PBMCs [44, 45]. DNA methylation differences in neuronal genes in PBMCs could reflect some central differences in psycho-neuroendocrine-immune pathways in ME/CFS, as suggested by our glucocorticoid sensitivity assay results, which aligns with previous work identifying peripheral blood and immune cells as suitable candidates reflective of DNA methylation differences in central systems [46–48].

### Dexamethasone response subgroups in ME/CFS

We observed a mean increase in glucocorticoid sensitivity in ME/CFS patients, which could not be explained based on type of ME/CFS onset or quality of life. In addition, a finer examination of our results revealed two subgroups among ME/CFS patients. Mild hypocortisolism and enhanced negative feedback to glucocorticoids were observed in several studies of GC responses in ME/CFS [3, 49]. The presence of both the GC-Typical and GC-Hypersensitive subgroups within our ME/CFS cohort thus aligns with the observed heterogeneity of HPA-related differences in these previous reports.

Glucocorticoids are known for their anti-inflammatory effects and are typically used to suppress immune responses. However, inappropriate response to glucocorticoid treatment is associated with increased susceptibility to metabolic and cardiac diseases [50]. Our results using PHA, a T cell mitogen, as an immune stressor indicate a functional impairment in T cell GR sensitivity in ME/CFS GC-Hypersensitive patients. Additional evidence suggests that T cells are candidates for a primary immune cell population in ME/CFS pathology. For example, a recent GWAS found significant differences in polymorphisms associated with T cell receptors in ME/CFS patients [51]. In addition, DNA methylation differences have been reported in CD4+ T cells from ME/CFS patients [52], a cell population that appears to show and increased dexamethasone sensitivity in ME/CFS [53].

We found 13 sites associated with glucocorticoid sensitivity in ME/CFS GC-Hypersensitive patients compared to both GC-typical ME/CFS patients and healthy controls. To our knowledge, no other EWAS or GWAS studies have specifically examined epigenetic or genetic differences in the context of GC sensitivity. However, genomic studies of ME/CFS have reported polymorphisms in GC signaling genes in ME/CFS patients. Interestingly, these genes do not appear to overlap with other disorders characterized by impaired GC signaling [51, 54]. In addition, FKBP5, a gene that was recently found to be differentially methylated in Cushing’s syndrome [55], was not among the genes identified in our study. At present, however, the potential link between ME/CFS and epigenetic modification of these genes should therefore be viewed with caution. Nevertheless, the results suggest that epigenetic differences at these sites may provide useful information regarding associated GC sensitivity among some ME/CFS patients.

Six of the 13 sites were part of known coding genes, four of which have roles in cellular metabolism. Patatin Like Phospholipase Domain Containing 4 (PNPLA4) is a phospholipase that plays a role in lipid metabolism and is highly expressed in metabolically active tissue [56]. PNPLA4 is also part of the PNPLA family, which activates upon glucocorticoid interaction [57]. D-aspartate Oxidase (DDO) is an enzyme that deaminizes D-aspartate and N-methyl D-aspartate, which is abundant in neuroendocrine tissue. Gene knockout studies of DDO in mice have revealed that this enzyme is important in melanocortin production [58] and involved in regulating basal corticosterone levels [59]. Phosphodiesterase 1C (PDE1C) is responsible for the hydrolysis of cyclic nucleotides, which is important for physiological regulation, calcium signaling pathways, and circadian rhythms [60]. Cell culture work has shown that inhibition of PDE1C via siRNA knockdown results in inhibited cell proliferation [61] and that PDE1C is activated upon dexamethasone treatment [62].

The top 3 sites, based on magnitude difference between ME/CFS GC-Hypersensitive and GC-Typical (ME/CFS and control) subjects, corresponded to GSTM1, MYO3B, and GSTM5, all of which showed >10% increase in methylation. GSTM1 and GSTM5 are part of the mu class of the GST gene family, whose primary role is the detoxification of environmental and exogenous toxins, specifically polycyclic aromatic hydrocarbons [63]. Genetic polymorphisms in GSTM are known to predict the potential response to glucocorticoid treatment in acute childhood lymphoblastic leukemia [64], indicating that GSTM may have a significant role in glucocorticoid signaling in immune cells. MYO3B is an ATPase that is activated by actin and is involved in kinase activity [65]. However, MYO3B and its various interactions remain poorly characterized compared to other myosin genes, making it unclear how differences in MYO3B may relate to glucocorticoid signaling.

The 13 differentially methylated sites could be considered to be biomarkers of glucocorticoid hypersensitivity, however additional work is required to understand and confirm the functional impact of hypermethylation on these genes and its relationship to glucocorticoid signaling. Gene knockout and RNA knockdown studies can assist in determining the precise impact that these genes have on glucocorticoid signaling. Measuring mRNA transcripts, methylation differences, and protein levels of these genes at baseline, PHA-stimulated, and DEX-suppressed conditions both in vitro and in vivo would provide a better understanding of the dynamics underlying GC sensitivity differences in ME/CFS.

### DNA methylation modifications associated with quality of life health scores

We found over 1600 differentially methylated regions that were significantly associated with overall RAND-36 score (Table 5; Additional file 10: Table S7), where variation in methylation at these particular regions was significantly associated with variation in the overall RAND-36 score. Scores from this survey may point towards alterations in biological systems. Notably, of the top 5 differentially methylated regions (Table 5), ATP6V0E2 (*R* ^2^ = 0.226) is an isoform of the H(+)-ATPase V0 e subunit, which is important for cellular energy [66], LOC401431 (*R* ^2^ = 0.224) encodes the antisense RNA for ATP6V0E2 suggesting that the regulation dynamics of this particular gene may be affected in ME/CFS, IL6R (*R* ^2^ = 0.220) encodes for the receptor of IL-6, a pleiotropic cytokine, and LOC144571 (*R* ^2^ = 0.220) is the antisense RNA to alpha-2-macroglobulin, a protease inhibitor and cytokine transporter. The low Physical Health scores in ME/CFS patients (Table 1) suggest that the physical impairment in ME/CFS is associated with an epigenetic imbalance of cellular energy, metabolism, and immune signaling.

## Conclusions

Here, we report DNA methylation differences in PBMCs of ME/CFS patients, some of which were significantly associated with overall quality of life as well as glucocorticoid hypersensitivity in a subgroup of ME/CFS patients. Notably, we determined epigenetic loci associated with differences in glucocorticoid sensitivity (Table 4) that may reflect underlying ME/CFS pathology in some patients. Additional work is required to confirm the potential mechanistic relationships between DNA methylation in these genes of interest, downstream gene expression and protein profiles, and ME/CFS phenotype. Longitudinal studies both in vivo and in vitro are needed to assess the stability of these epigenetic modifications, including changes in symptom profiles and in response to glucocorticoid treatment. For example, cytokines such as IL-10 and IFN-gamma, which were differentially methylated in our study (Additional file 7: Table S4), are known to interact with GR and show expression differences in vitro upon dexamethasone treatment [53, 67]. While GR density and binding affinity in ME/CFS PBMCs do not appear to differ in steady state conditions [4], GR is known to be upregulated during exercise challenge in ME/CFS [68]. Future work should examine DNA methylation signatures during exercise challenge in ME/CFS in order to gain a better understanding of glucocorticoid signaling dynamics. Nevertheless, at the very least, the differentially methylated sites identified in this study may be important as biomarkers for future clinical testing in order to determine if epigenetic changes in these genes associate with disease onset or progression.

The results of this study highlight the potential utility of immune cell subtyping within the ME/CFS population, and indicate that epigenetic data may aid in elucidating relevant biological pathways impacted by ME/CFS. Clinical investigations of the regulation of cellular metabolism are needed to assess this possibility, as we found that genes such as GSTM1, MYO3B, GSTM5, and ATP6V0E2 showed significant epigenetic modifications in ME/CFS. Increased understanding of ME/CFS subtypes will assist patients and physicians to determine the appropriate interventions to treat symptoms and improve personal health.

## Additional files

Additional file 1: Figure S1. PCA results of RAND-36 scores. (A) Principal components (PCs; columns) and the amount of variance each PC accounts for in the data (B) RAND-36 principal component scores of PC1 against PC2 for ME/CFS (red) and control (green) subjects. Loadings for (C) PC1 and (D) PC2 are separated according to the 8 different categories of RAND-36. (TIF 161 kb)
Additional file 2: Table S1. Differentially methylated sites in ME/CFS. Sites that were significantly differentially methylated between ME/CFS and controls (FDR ≤ 0.05, Beta-difference ≥ 5% ). (XLSX 1.58 mb)
Additional file 3: Figure S2. Distribution of differential methylation in ME/CFS. The distribution and genic locations of differentially methylated loci in ME/CFS patients compared to healthy controls (*n* = 12,608). (A) The proportion of probes that are either hypomethylated (decreased methylation, white) or hypermethylated (increased methylation, black) in ME/CFS patients. The proportion of hypo- and hypermethylated probes according to (B) genic regions or (C) location relative to CpG islands are displayed. Line above bars = Main effect of region, * = *p* <0.05, Pearson Chi-Squared test. * = post-hoc FDR < 0.05, comparison with CpG island, unless indicated by hooked lines. (TIF 219 kb)
Additional file 4: Table S2. High confidence differentially methylated sites according to Dexamethasone assay subgroups. Differentially methylated sites between the 3 different subgroups (ME/CFS GC-Hypersensitive, ME/CFS GC-Typical, and Healthy Controls) from the Dexamethasone assay (*p* ≤0.05, Beta-difference ≥ 5%, after 10 000 permutations). (XLSX 2.88 mb)
Additional file 5: Table S3. Differentially methylated sites that relate to GC sensitivity in ME/CFS. Full annotation of 13 sites that relate to GC sensitivity in ME/CFS GC-Hypersensitive subjects (*p* ≤0.05, Beta-difference ≥ 5%, after 10 000 permutations). (XLSX 48.9 kb)
Additional file 6: Figure S3. Permutation results of GC sensitive sites. Beta-difference distributions of the 13 sites potentially associated with GC sensitivity after 10,000 permutations, according to (A) ME/CFS GC-Hypersensitive vs. ME/CFS GC-Typical, (B) ME/CFS GC-Hypersensitive vs. Controls, and (C) ME/CFS GC-Typical vs. Controls. The red line in each panel indicates the location of the distribution where *p* <0.05 and the blue line indicates the observed beta-difference on the 450 K array. (TIF 3.44 mb )
Additional file 7: Table S4. High confidence epigenetic biomarkers for ME/CFS. Epigenetic loci that relate to general ME/CFS pathology (*p* ≤ 0.05, Beta-difference ≥ 5%, after 10 000 permutations). (XLSX 543 kb)
Additional file 8: Table S5. Gene ontology (GO) terms associated with differential methylation in ME/CFS. GO terms of the sites associated with ME/CFS (FDR ≤ 0.10). (XLSX 74.8 kb)
Additional file 9: Table S6. Differentially methylated sites unique to ME/CFS GC-Typical. Epigenetic loci that are specifically differentially methylated in the ME/CFS GC-Typical subgroup (*p* ≤ 0.05, Beta-difference ≥ 5%, after 10 000 permutations). (XLSX 24 kb)
Additional file 10: Table S7. Epigenomic regions that are significantly related to quality of life. Genes and associated regions that showed significant associations between RAND-36 overall quality of life and DNA methylation (FDR ≤ 0.05). (XLSX 168 kb)

## Abbreviations

450 K: Illumina infinium humanMethylation450 beadChip
BMI: Body mass index
BrdU: Bromodeoxyuridine
DMSO: Dimethyl sulfoxide
DPBS: Dulbecco’s phosphate-buffered saline
ELISA: Enzyme-linked immunosorbent assay
FBS: Fetal bovine serum
FDR: Benjamini-Hochberg procedure/false discovery rate
GC: Glucocorticoid
GEO: Gene expression omnibus
GO: Gene ontology
GR: Glucocorticoid receptor
GST: Glutathione-S-transferase
HPA: Hypothalamic-pituitary-adrenal
IMA: Illumina methylation analyzer
ME/CFS: Myalgic encephalomyelitis/chronic fatigue syndrome
PBMCs: Peripheral blood mononuclear cells
PCA: Principal component analysis
PCR: Polymerase chain reaction
RAND-36: The RAND 36-item health survey
RPMI-1640: Roswell park memorial institute 1640 medium
SNP: Single nucleotide polymorphism
SWAN: Subset-quantile within array normalization
TSS: Transcription start site
UTR: Untranslated region
