# Supplementary figures and images for: Epigenetic modifications and glucocorticoid sensitivity in Myalgic Encephalomyelitis/Chronic Fatigue Syndrome (ME/CFS)
Source: BMC Med Genomics. 2017 Feb 23;10:11. doi: 10.1186/s12920-017-0248-3 (PMC5324230; doi:10.1186/s12920-017-0248-3)

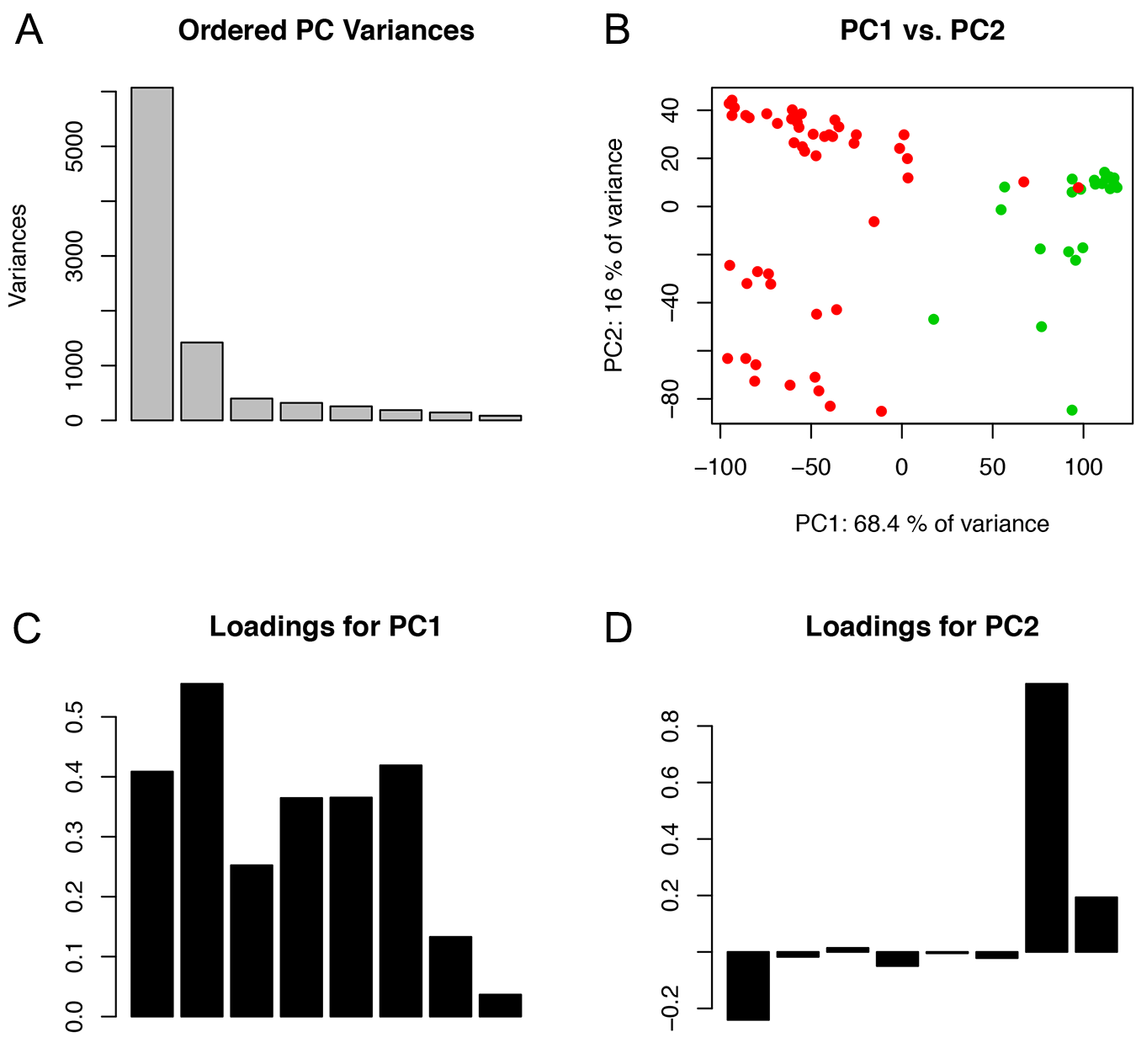

Supplement: Additional file 1: Figure S1. — PCA results of RAND-36 scores. (A) Principal components (PCs; columns) and the amount of variance each PC accounts for in the data (B) RAND-36 principal component scores of PC1 against PC2 for ME/CFS (red) and control (green) subjects. Loadings for (C) PC1 and (D) PC2 are separated according to the 8 different categories of RAND-36. (TIF 161 kb) [file 12920_2017_248_MOESM1_ESM.tif]

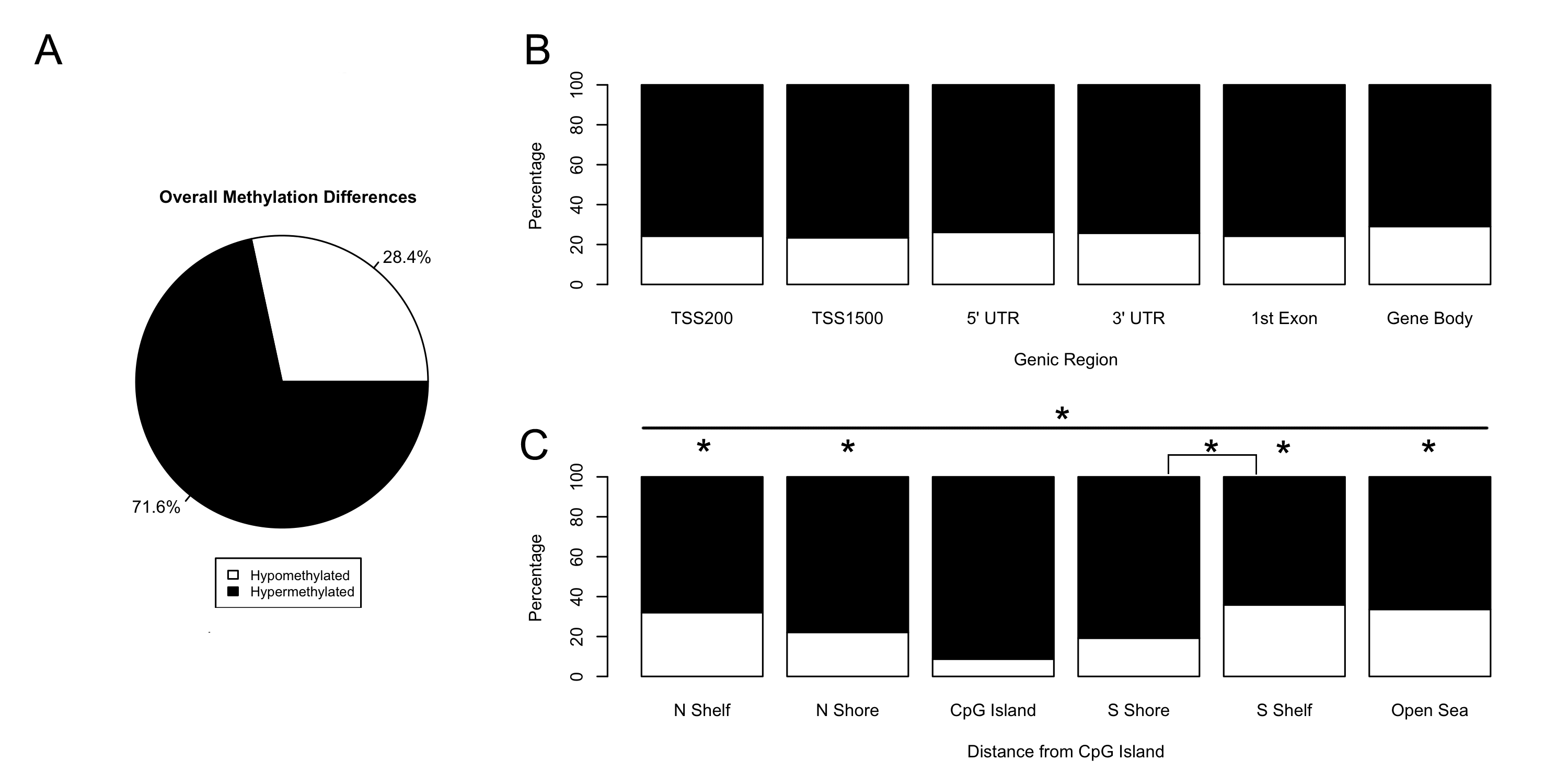

Supplement: Additional file 3: Figure S2. — Distribution of differential methylation in ME/CFS. The distribution and genic locations of differentially methylated loci in ME/CFS patients compared to healthy controls (n = 12,608). (A) The proportion of probes that are either hypomethylated (decreased methylation, white) or hypermethylated (increased methylation, black) in ME/CFS patients. The proportion of hypo- and hypermethylated probes according to (B) genic regions or (C) location relative to CpG islands are displayed. Line above bars = Main effect of region, * = p <0.05, Pearson Chi-Squared test. * = post-hoc FDR < 0.05, comparison with CpG island, unless indicated by hooked lines. (TIF 219 kb) [file 12920_2017_248_MOESM3_ESM.tif]

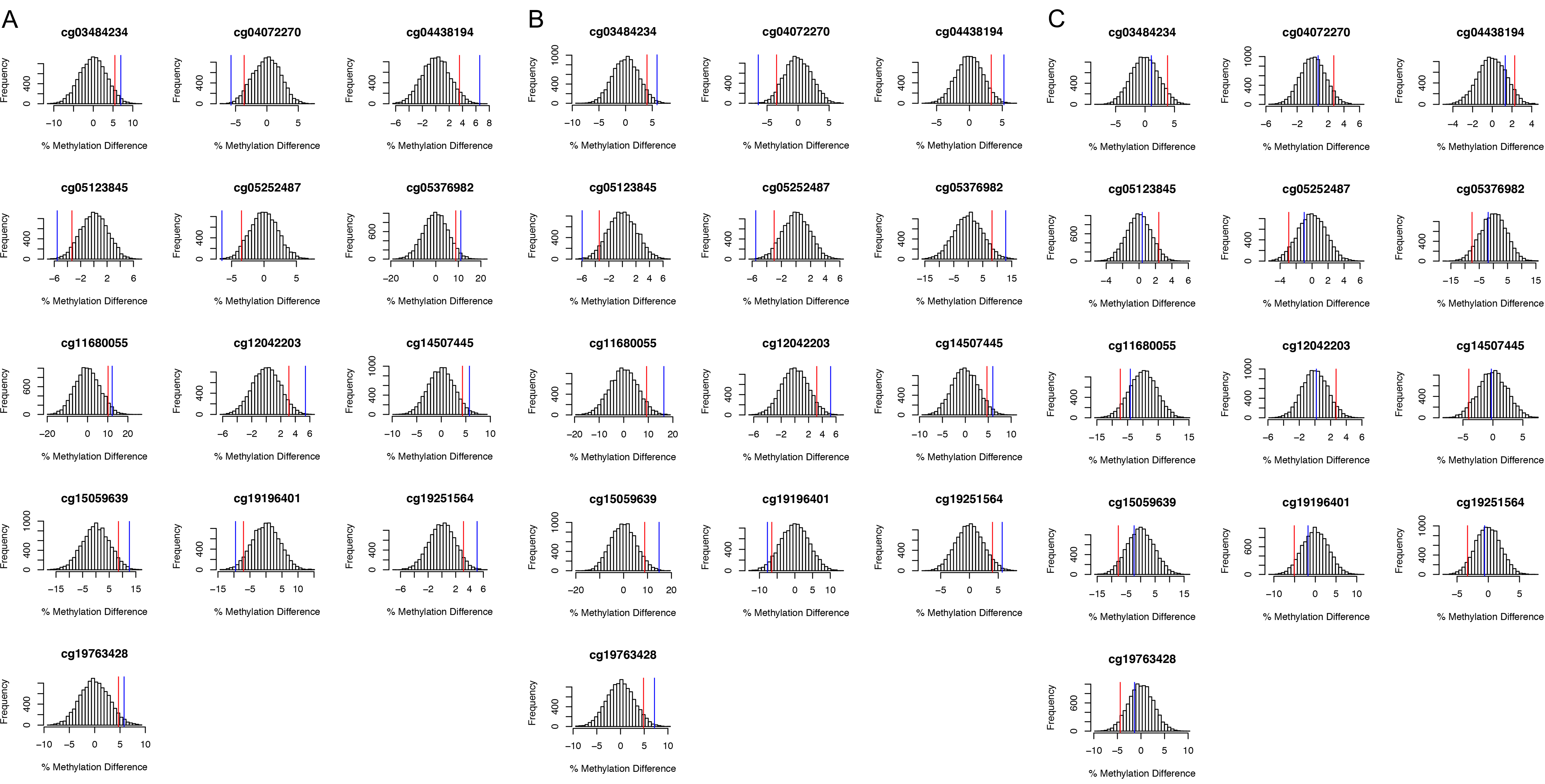

Supplement: Additional file 6: Figure S3. — Permutation results of GC sensitive sites. Beta-difference distributions of the 13 sites potentially associated with GC sensitivity after 10,000 permutations, according to (A) ME/CFS GC-Hypersensitive vs. ME/CFS GC-Typical, (B) ME/CFS GC-Hypersensitive vs. Controls, and (C) ME/CFS GC-Typical vs. Controls. The red line in each panel indicates the location of the distribution where p <0.05 and the blue line indicates the observed beta-difference on the 450 K array. (TIF 3.44 mb ) [file 12920_2017_248_MOESM6_ESM.tif]
